# Parent-Focused Psychotherapy for the Preventive Management of Chronicity in Anorexia Nervosa: A Case Series

**DOI:** 10.3390/ijerph19159522

**Published:** 2022-08-03

**Authors:** María García-Anaya, Alejandro Caballero-Romo, Laura González-Macías

**Affiliations:** 1 Clinical Research Division, National Institute of Psychiatry Ramón de la Fuente Muñiz, Mexico City 14370, Mexico; 2 Eating Disorders Clinic at Clinical Services Division, National Institute of Psychiatry Ramón de la Fuente Muñiz, Mexico City 14370, Mexico; caballer@imp.edu.mx

**Keywords:** anorexia nervosa, family psychotherapy, chronicity, reflective functioning, vicarious experience

## Abstract

Background: Anorexia nervosa (AN) is a complex eating disorder where involvement of family plays a central role in first line treatment in adolescents, but which is not so for adults where poor response to treatment is frequent. Given the reluctance of some patients to receive treatment, we set out to explore the hypothesis that certain family dynamics may be involved in the maintenance of the disorder. Methods: We aimed to understand what is underlying in the cases of patients who present clinical improvement with their parents, but not the ones who received a parent-focused psychotherapeutic intervention. We conducted a mixed methods study. On the one hand we performed a case series of 14 patients who dropped out of treatment while their parents actively attended the intervention, and on the other hand, we followed the evolution of the parents of those patients reluctant to continue treatment, through non-participant observation. Results: We present preliminary evidence where we found the parent-focused psychotherapeutic intervention was able to elicit a reflective function of the parents. We also observed that the intervention modified certain family dynamics that could be related to maintaining factors of the disorder. In patients, we found that in parallel to the assistance of their parents to psychotherapeutic treatment, and even when they were receiving no intervention, they showed significant clinical improvement of symptomatology and global functioning; we observed 9 of 14 of them who voluntarily decided to return to pharmacological treatment. Conclusions: This parent-focused intervention elicited changes in reflective functioning of participant parents; the intervention produced favorable changes in family dynamics, which we believe is probably related to improvement of global functioning, symptomatology, and insight of patients.

## 1. Introduction

Anorexia nervosa (AN) is an eating disorder characterized for: (a) restriction of energy intake relative to requirements, (b) intense fear of gaining weight or becoming fat, (c) disturbance in the way in which one’s body weight or shape is experienced. Given its main manifestations, it could be subclassified as restricting type or binge-eating/purge type [1]. A recent review reported a lifetime prevalence rate ranged between 0.1% and 3.6% in females and 0.0% and 0.3% in males [2].

AN elicits the severest consequences among eating disorders and is the only psychiatric entity that, like other serious medical conditions, may per se lead to death due to deterioration of the general condition [3,4,5,6]. It constitutes a disorder with a highly variable clinical course, elevated rates of poor response to treatment, and mortality rates among the highest of any psychiatric disorder [7,8,9]. Current clinical guidelines point out family-based interventions as the first line of treatment for adolescents, showing good results [8,10,11]. This is not so in the adult population, for whom results have only been moderately effective and the family is not always addressed by the treatment [12,13,14]. Among all treatment options, we find diverse theoretical postures and specific psychotherapeutic schemes according to diagnosis, age, and stage of disorder [8,10,15,16,17,18,19,20,21].

Despite the effectiveness of these treatment programs, an important percentage of individuals whether remaining unresponsive to treatments or responding who later relapse. In this sense, statistics have shown that approximately 30% of persons with AN, improve after standard treatment but still experience symptoms that continue affecting their functioning, and approximately 20% of them remain chronically ill [7]. High rates of relapse have been reported, long-term prognosis is often poor [22], and specifically in adults, mortality rates of between 9% and 20% have been observed over 12-to-20-year follow-up periods [23,24]. Moreover, persons with AN are frequently characterized for showing high rates of treatment denial, treatment avoidance, and treatment dropout [25,26], which drastically impairs their prognosis, and raises the likelihood to suffer serious medical consequences [27,28] and develop a severe and enduring disorder [29]. Additionally, people with chronic AN exhibit a long-term impairment of the overall functioning equivalent to that displayed in other severe and chronic mental disorders such as schizophrenia [7,30].

Our experience with this population at the Clinic for Eating Disorders (CED) of the National Institute of Psychiatry Ramon de la Fuente Muñiz in Mexico City, has shown us that around 20–30% of patients remain unresponsive to treatment or present poor response and subsequent relapse, even after receiving standard treatment for sufficient time and dose, according to treatment guidelines [31,32,33]. For those patients, our treatment algorithm contemplates *monitoring alongside a parent-focused psychotherapy group*. Monitoring involves two elements: (1) Providing general support to patients; comprising pharmacotherapy, appointments with the psychiatrist every 6 to 12 weeks, and attendance with the group of patients at risk of chronification; (2) Working with the patient’s family through a psychotherapy group for parents (PGP). Unfortunately, a frequent scenario we face with these patients, is reluctance to undergo treatment (psychotherapy group and medications). In contrast, most parents of these patients are willing to continue at the PGP regardless of the reluctance of their daughter/son to continue treatment. We were able to empirically observe how daughters/sons of parents who continued to attend the PGP, progressively improved their global functioning and symptomatology, even when they dropped out of treatment [34]. This made us think that some changes, in terms of the reflective function [35] of parents may be contained in this phenomenon.

From these observations, we posed as a hypothesis that there are certain family dynamics that, in some cases, promote maintenance of factors of the eating disorder, so working with parents to observe, identify, and modify behaviors implicated in the way they relate as a family, will foster clinical and functional changes in patients. Our hypothesis and the PGP′ design are held on the theoretical foundations and clinical observations that Salvador Minuchin, Mara Selvini-Palazzoli and Hilde Bruch made about family functioning in AN. Minuchin reported that eating disorders occur in the context of family dynamics that always follow the same rules, so a maintenance circuit emerges and the psychosomatic symptom is auto-perpetuated over time [36]. Bruch revealed the existence of conflicts related to parental attitudes towards their child’s disorder, and to the quality of attachment of primary caregivers [37,38]. Selvini-Palazzoli pointed out that each member of the family follows an individual strategy to achieve their own objectives and to participate in the ongoing game within the family [39,40]. More recently, the contribution of these family aspects has also been recognized and addressed in patients and some of their relatives by the Maudsley model from an interpersonal cognitive model [41,42].

We believe that what is behind our empirical observations has great potential as a therapeutic alternative to address—through parents willing to receive an intervention—those cases at risk to evolve to chronicity, given their bad response to standard treatment and their refusal to continue with it.

The aim of this article is to present preliminary evidence of the empirical data we collected and analyzed in order to understand what underlies the clinical improvement of patients who declined to continue treatment, but whose parents received it instead, for a case series of 14 patients and their parents. We also aimed to describe the main features of this intervention that holds an integrative perspective [43], designed at the CED and not previously exposed.

## 2. Materials and Methods

### 2.1. Population, Inclusion Criteria, and Methodology

**Figure 1:**
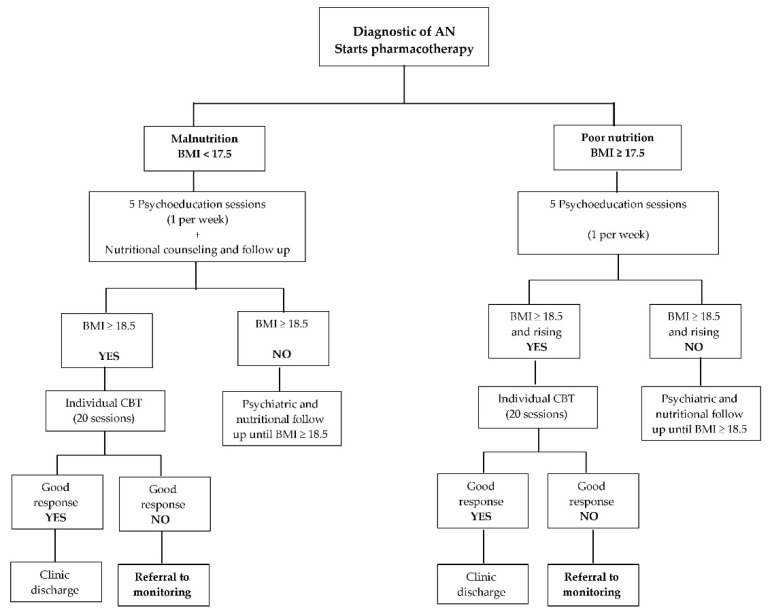
Algorithm of treatment used at the CED. Time elapsed from admission to the clinic to referral to monitoring, varies depending on each patient’s individual response, number of relapses, and adherence to treatment.

We conducted a mixed methods study. The quantitative part of the study comprises a case series for which we made an in-depth review of the clinical records of 14 patients to monitoring. As for the qualitative part, we performed a follow up of the psychotherapeutic process of their parents at the PGP. Regarding the case series as our inclusion criteria, we only reviewed in-depth the cases of patients who remained unwilling to receive pharmacotherapy and psychotherapy and, in parallel, their parents actively attended the PGP. To track each patient evolution from the clinical records, we documented their clinical picture regarding symptomatology and overall functioning at three points: (1) upon admission to the CED, (2) at the reference to monitoring—in Table 1 we indicate how many months after having followed the treatment algorithm (Figure 1), patients were sent to monitoring—and (3) at the end of the follow-up—up to 18 months after monitoring started. Besides symptomatology, we considered body mass index (BMI) and global functioning (assessed by Global Assessment Functioning scale, GAF), as two cardinal signs of change of the patients’ clinical condition. We performed a t-paired test to compare the BMI and GAF scores at the reference to monitoring (which is when parents began attending the PGP), and at the end of the follow-up. Since these cases were considered difficult to manage and at high risk of becoming chronic, their evolution was collegially evaluated and discussed by the multidisciplinary team of the CED—made up of 4 psychiatrists, a psychotherapist, and a nutritionist, all of them experts in eating behavior disorders—at the weekly meetings of the clinic.

As for the qualitative part of the study, we used non-participant observation to follow and understand the parents’ narratives through the therapeutic process, and to characterize the intervention. We also extracted from the observation sheets the in vivo codes with narratives that reflect changes in the evolution of parents in a positive sense. To follow up parents’ evolution, we made a cross reference between the narratives from the observation sheets and the therapists’ notes. Non-participant observation also allowed us to identify and define the structure, dose, therapeutic style, and theoretical foundations of the intervention.

The intervention and procedures we present here, have been delivered at the CED of the National Institute of Psychiatry Ramon de la Fuente Muñiz for the last 20 years. In Table 1, we show the treatment algorithm applied at the CED, which comprises standard treatment (ST). All patients undergo this algorithm and the time elapsed from admission to the clinic to referral to monitoring, varies depending on each patient’s individual response, number of relapses, and adherence to treatment. All patients included here were referred to monitoring either during a relapse or at impasse, but always after having undergone the algorithm.

Determination of clinical and cognitive level impasse is a collegiate assessment made by the multidisciplinary team of experts of the CED, based on the clinical evaluations of symptomatology and global functioning they previously made of each patient.

For standard treatment we refer to comprehensive management with psychiatric, nutritional, psycho-educative, and psychotherapeutic care according to the APA and NICE guidelines [31,32,33].

### 2.2. Characteristics of the Intervention

- Dose of the intervention

This is an open group working under a multilevel scheme, where novel and veteran parents co-participate and share their experiences relative to their daughter’s/son’s eating disorder. Group sessions are held once a week for 2 h.

- Group structure

The group is structured observing the eleven specific therapeutic factors identified by Vinogradov and Yalom [44] as therapeutic mechanisms operating in group psychotherapy: (1) instilling hope, (2) universality, (3) transmitting information, (4) altruism, (5) development of socialization techniques, (6) imitative behavior, (7) catharsis, (8) corrective recapitulation of the primary family group, (9) existential factors, (10) group cohesion, (11) interpersonal learning.

- Therapeutic style

This is an intervention with an integrative psychotherapy perspective. It uses as framework a structural systemic foundation, and combines psychodynamic, cognitive analytical [45], and supportive psychotherapy techniques [46,47]. The group is facilitated by one psychotherapist trained as a psychoanalyst in cognitive-behavioral therapy; and two co-therapists trained as psychiatrists specialized in eating disorders.

- Structure of the sessions

At the beginning of the group sessions, the psychotherapist allows free speech about concerns or recent events related to their daughter/son’s disorder; from the information exposed by the attendees, the psychotherapist detects and defines a theme for the session. From this theme, the psychotherapist points out parental functions that are at stake. Then he/she gives the floor to participants inviting them to provide feedback based on their own experience; veteran parents speak first, and novel parents later (this rule emerged naturally in the group dynamic). During feedback, experience and learning acquired through this process are transmitted from parent to parent. The psychotherapist contains a dialogue between pairs, provides clarification and empathic validation, stimulates insight, gives psychoeducation, interprets, and metabolizes information. He/She always uses relapse as the context to observe what, how, and why they function at four levels: individual, familiar, couple relationship, and father-daughter/mother-daughter dyad. Afterwards, once the feedback reaches a saturation level, the psychotherapist operationalizes the material discussed in the group from the biopsychosocial model, highlighting the identified functioning linked to maintaining factors of the disorder. Finally, the psychotherapist works out a conclusion of the session oriented towards the participants reflection over the individual behaviors that promote the maintenance of the disorder.

- Topics emerged in sessions

We observed, until reaching the saturation of the information, that the topics parents exposed as concerns in the session were as follows: difficulties to set limits, difficulties to emotionally contain their daughter/son, difficulties to cope with their own emotions and those of their daughter/son, difficulties to be in authority, parenting styles, children as buffer of the couple relationship, giving responsibility back to their daughter/son, mistrust of their daughter/son, concerns about the way of eating of daughter/son, fear of losing their daughter/son.

## 3. Results

### 3.1. Demographics

We explored 14 individuals diagnosed with AN. Of them, 12 were female (86%) and two were male (14%); their ages upon admission to the CED ranged between 17 and 30 years old (mean = 20.2 years), and we found diagnostic variations where 10 individuals had binge/purging type (BP) and four had restrictive type (R).

### 3.2. Clinical Records

From the in-depth review of clinical records, we found the time to referral of patients to monitoring after receiving standard treatment ranged between 4 to 16 months (mean = 7.7 months). Time from the beginning of monitoring to the end of follow up ranged between 6 to 18 months (mean = 13.8 months).

**Figure 2:**
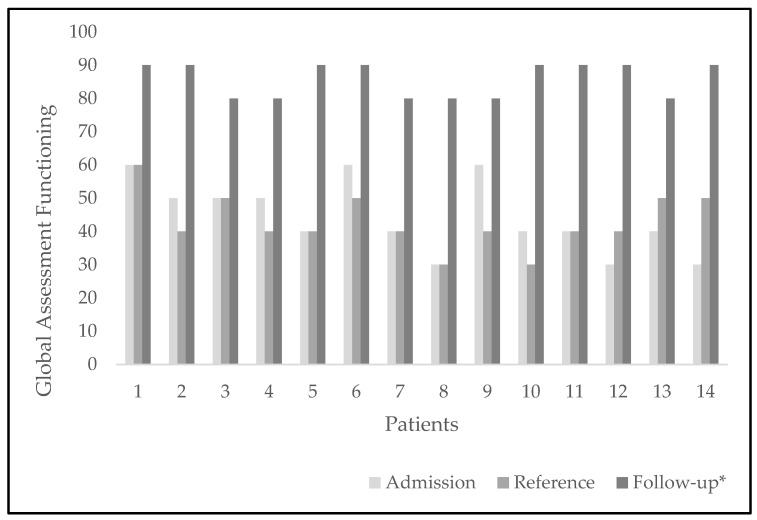
Global assessment functioning of every patient with time. Comparison of GAF values at three points: upon admission to the CED, at reference to monitoring, and at the end of follow-up. * GAF scores were significantly higher at follow-up, than at referral to monitoring, *p* = 0.0009.

Each patient displayed a particular set of symptoms but regarding global functioning (GF), patients either impaired or remained unchanged from admission to CED in reference to monitoring. Six from14 patients showed the same values scored by GAF, while in eight of 14 patients GAF values were lower that the basal ones. At follow-up assessment, all patients showed improvement of GF, reaching values over 80 points (Figure 2). We found statistically significant differences when contrasting the GAF values, where the scores at the time of referral to monitoring (M = 42.8; SD = 8.2) were lower than the scores at the end of follow-up (M = 85.7; SD = 5.1) t_(13)_ 17.5, *p* = 0.0009.

**Figure 3:**
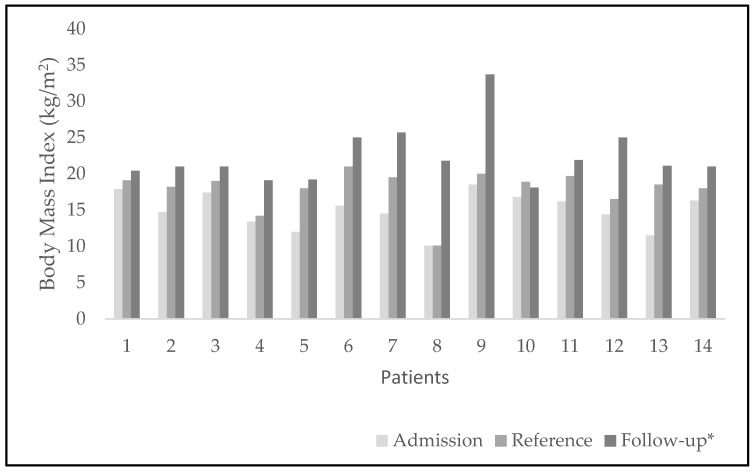
BMI of every patient with time. Comparison of values at three points: upon admission to the CED, at reference to monitoring, and at the end of the follow-up. * BMI scores were significantly higher at follow-up, than at referral to monitoring, *p* = 0.002.

We also observed BMI increased from admission to reference in all patients from underweight to healthy weight in seven patients; the other seven patients presented increases in BMI values as well, though remaining in underweight levels (Figure 3). We found statistically significant differences when contrasting the BMI values, where the scores at the time of referral to monitoring (M = 18.5; SD = 1.5) were lower than the scores at the end of follow-up (M = 22.0; SD = 2.8) t_(13)_ 6.04, *p* = 0.002.

However, despite the weight gain, in the same period all of them entered into impasse at a cognitive level and showed no insight about the eating disorder. By the end of the follow-up, dietary patterns, GF, and insight of all patients improved (Table 1).

### 3.3. Demographics

We followed the parents of 14 patients: 13 women and 7 men. They made up a total of 20 individuals, given that only in six cases the couple attended the PGP together. Among them, 13 subjects remained married and were living as a couple, and one of them was divorced. All these parents attended the PGP for at least one year, and they completed a minimum of 40 sessions.

### 3.4. Follow Up of Parents

We observed, identified, and analyzed—upon reaching saturation of information—parents’ narratives around their daughter/son’s eating disorder related to the way it impacted their behaviors, beliefs, and their internal representations of the family and of each family’s integrant.

Regarding the evolution of the parents, we present here four examples with in vivo codes extracted from the non-participant observation and the therapists’ notes, where we illustrate interactions that frequently occurred in the sessions and where we were able to identify the three different points of the process.

Example 1: A novel mother speaks about her concern because her daughter is still not eating.

“*I don’t know what to do, I prepare fruit for her for breakfast when she leaves home and when I come back home, I see the lunchbox in the kitchen with the fruit intact.*”

The psychotherapist asks her:

“*And why do you think she brings the fruit back home?*”

The mother answers:

“*Because she is still afraid of gaining weight.*”

The psychotherapist asks the mother:

“*If your daughter didn’t want you to notice that she doesn’t eat the fruit you prepare for her, do you think she would bring it back? Wouldn’t it be easier for her to throw it out there, and avoid being scolded?*”

This novel mother remains silent, so the psychotherapist asks veteran parents to provide feedback. A veteran mother says:

“*The same thing used to happen to me, until I understood that my daughter wanted to get my attention, and I started communicating with her. What your daughter wants is not to stay thin, but for you to pay attention to her.*”

Example 2: A novel mother shares an episode where she argued with her adult daughter (24-year-old) because she does not want to eat, so asks for advice on how to get her daughter to eat without fighting.

The psychotherapist asks the mother:

“*Why do you think your daughter does not want to eat?*”

The mother answers:

“*I think she is upset with me; when the food isn’t hot and the table isn’t set, she doesn’t want to eat.*”

The psychotherapist asks veteran parents for feedback. Another mother says:

“*I think you are right; your daughter is upset but not because the meal was not ready. I have experienced the same, I used to do everything for her, even her school homework, but you know what, that’s like telling them, you’re useless, you can’t. They are angry but they don’t say it, they act it.*”

Then the psychotherapist clarifies:

“*So, maybe your daughter does not eat because she knows that distresses you, don’t you think so?*”

Example 3: A mother reveals she knows her daughter is consuming cannabis and doesn’t know how to get her to stop smoking. About it she says:

“*I think I have been too soft with my daughter.*”

The psychotherapist asks her:

“*Have you really been soft? Because you just told us you’ve being checking her cell phone and snooping her purse and drawers. That rather seems like you are controlling, don’t you think so?*”

The mother says:

“*Yes, it’s true and I don’t know how to do it, I think I’ve always lived in the extreme, I don’t know where the middle is” … “I realize what my daughter is into, but I turn a blind eye, although afterwards sadness and anger float in the air and I act out the emotion instead of speaking it.*”

Example 4: A father talks about his concern about miscommunication with his daughter. He points out that he tries to be interested in her and he looks for small talk, but he has noticed that many times she avoids him or leaves him talking alone in the middle of a conversation. He asks for advice to improve communication.

“*Before I didn’t pay attention to her and now that I try to talk to her, she avoids me. I understand that communication can’t be built in a second, but I don’t know what to do because no matter how hard I try, she is not interested.*”

The psychotherapist says:

“*Has anyone been through something similar and would share their experience?*”

A veteran father says:

“*My wife and I have talked about just that, because our daughter did the same. We thought we were laid back and accessible, but we gave too much importance to her being the best in school, to always looking how we wanted. We made seemingly subtle comments to her like: you should wear more cheerful clothes, or why do you like that kind of music, or you stand out because you are the best in your school-group. And that is not accepting and respecting your daughter. Now we understand that she felt it as a very strong pressure.*”

The psychotherapist says:

“*What he just said is related to our expectations as parents. We must observe, what idea do I have of my daughter? How do I think she should be? So, we may be able to distinguish the difference between the daughter that I want and the daughter that I have, and then relate with the real needs of the daughter.*”

We operationalized the three points of the process as:

(1) **The phase of the maintainers.** Where parents are only focused on the eating behavior and the compliance of treatment.
  “*I don’t know what to do, I prepare fruit for her for breakfast when she leaves home and when I come back home, I see the lunchbox in the kitchen with the fruit intact.*”
  “*I think she is upset with me; when the food isn’t hot and the table isn’t set, she doesn’t want to eat.*”
(2) **The phase of emergence of reflective function (epiphany).** Where parents stopped concerning about amount and type of food and started observing the reasons why their daughter/son has this kind of relationship with food. At this point we observed that reflective function begins to emerge towards the eating disorder, so we could see how even when parents did not know how to work out the situations, they began talking about their beliefs, emotions, and behaviors around the eating disorder, so they could observe how they feel and act in relation to it.
  “*Yes, it’s true and I don’t know how to do it, I think I’ve always lived in the extreme, I don’t know where the middle is” … “I realize what my daughter is into, but I turn a blind eye, although afterwards sadness and anger float in the air and I act out the emotion instead of speaking it.*”
(3) **The phase of consolidation of reflective function.** Where we observed how reflective function allows them to identify how their behaviors and emotions promote the maintenance of the eating disorder, and they understand what they must do to stop functioning that way.
  “*The same thing used to happen to me, until I understood that my daughter wanted to get my attention, and I started communicating with her. What your daughter wants is not to stay thin, but for you to pay attention to her.*”
  “*… We made seemingly subtle comments to her like: you should wear more cheerful clothes … And that is not accepting and respecting your daughter. Now we understand that she experienced it as a very strong pressure.*”

## 4. Discussion

Given the severe consequences that anorexia nervosa carries, we find it of great importance to evaluate the preliminary evidence we present here, and to propose the intervention that may potentially reduce chronification among this population.

We could identify how despite all patients gaining weight and improving their dietary patterns from admission to CED to the moment they were remitted to monitoring, they all remained in impasse at a cognitive level which in our perspective is the element that elicited impairment of symptomatology and GF as well. Subsequently, we find of interest how at the follow-up phase, all individuals showed remission of symptomatology and improvement in GF, even when it was their parents and not them who remained in the intervention. We think this effect is related to the process experienced by parents during the intervention in the PGP.

We want to highlight that the PGP framing sets as primary outcome the improvement of GF, just as other groups have done [29] and we set regaining weight and maintenance of a regular eating pattern as secondary goals. Since all patients had unfortunately failed to achieve goals related to maintaining a healthy food intake and body weight, we pursued for these patients, through their parents, the achievement and maintenance of personal characteristics that are more in line with the recovery model [29,48].Observing the group, we could learn how the main concern of novel parents is the way and amount their daughter/son’s eat, and as they become veterans their preoccupations about food are reduced, and the main concern turns into the way their daughter/son’s relate to them as parents and family. We observed that the transition from one stage to another is given by the understanding of how eating behavior works as a communication channel between parents and their daughter/son, and where the difficulties of exerting parental skills to interpret and facilitate understanding and communication of emotions are manifested.

We noted the way parents moved from one stage to another which occurred through two main elements: vicarious learning [49], and emergence/enhancement of reflective function [35]. On the one hand, we observed an effect of mirroring among participants, where veteran and novel parents’ interaction promoted a vicarious experience, so learning, understanding, and awareness are accelerated, and transmission of therapeutic and psycho-educative information is facilitated, as has been somewhat described before [50]. During the sessions we were able to systematically witness how when the psychotherapist clarified thoughts and behaviors they had normalized about their daughter/son’s disorder, parents usually showed hesitancy or skepticism to the psychotherapist comments, rationalizing and justifying the way they think and act. However, when other parents, usually the veteran ones, commented about the situation just exposed, novel parents frequently showed more receptivity, progressively interested in deepening their knowledge and even grateful for the feedback. On the other hand, we could see that in parallel to vicarious learning, the psychotherapist underpins a reflexive function, by facilitating the understanding of the communication style that the family handles, and the function that eating behavior has for them as a communication channel. We found that the psychotherapist delivers an intervention with an integrative psychotherapy perspective, from an assimilative integration approach, which in our case implies working primarily from within a psychodynamic model but integrating aspects of the other (mentioned above) when needed [43,51,52].

The psychotherapist works from an expressive, cognitive, and supportive style to guide the sessions. Using a psychodynamic style [53], the psychotherapist fosters a reflective function of the participants, so they are able to understand behaviors, emotions, and thoughts helping to maintain the familiar functioning underlying the disorder. Reflective function is also enhanced by cognitive analytical interventions [45] that facilitate identification of the cognitive scheme (thoughts, beliefs, emotions) and the style of individual and family functioning that enables maintenance of the disorder. Administration of supportive psychotherapy techniques [46,47] help the psychotherapist focus on self-esteem and adaptive skills, as well as supporting comforting, encouraging, containment, and limit setting. The psychotherapist also fosters growth, autonomy, and self-sufficiency. As we stated above, we consider, vicarious learning acts as a therapeutic enhancing element of reflective function which is of course sustained on the guidance, containment, and input of the psychotherapist.

We believe the development/enhancement of reflective functioning of parents is the element underlying changes in GF, symptomatology, and insight of patients. Mentalization, as the ability to signify the experience of oneself and others, enables in individuals elaboration of a mental state where cognitive processes and affective regulation take place [54,55]. We could witness how throughout the sessions, parents developed the ability to identify and understand the reasons underlying their daughter/son’s behavior, which enabled them to transform the way they relate to her/him. Likewise, we were able to see how strengthening parent’s reflective function was the key element for them to recognize and understand their own beliefs, emotions, and behaviors regarding the experience of having a daughter/son with an eating disorder [35]. In that sense, while working through the topics that emerged in the session, parents delved into the knowledge and insight of their own participation in family dynamics that contributed to maintenance of the disorder. As a possible related consequence of the changes elicited by this intervention on family dynamics, interestingly, we were able to see how 9 out of 14 patients, decided voluntarily to reinitiate pharmacological treatment when their parents were still working at the PGP by the end of the follow-up phase.

Just as we have pointed out the family dynamics that underlie the maintenance of the eating disorder, we acknowledge there are different coping strategies [56,57,58] and communication styles [59,60] affecting the way family members interact and relate to each other, however we did not delve into them since the study of those factors exceeds the objective of this article.

Nevertheless, just as certain familiar types of functioning constitute a maintaining factor of the disorder; in another sense it is also the family who plays a central role in shaping an individual’s well-being [61]. We acknowledge AN is the result of a combination of biopsychosocial factors and certainly the above-mentioned maintaining factors are not present in all families of individuals with AN. We understand, this intervention will not fit every case of AN with previous poor response to standard treatment, however we believe it is worth exploring the elements present in family dynamics as possible maintenance factors on which we may impact and prevent chronification. Furthermore, we consider this intervention could be a valuable therapeutic resource for those cases where patients show reluctance to continue treatment, but their parents are willing to continue with an intervention that may help them understand and manage their daughter/son’s disorder.

We are aware that the results presented here are quite preliminary and may not be generalized to the population with AN and who have had a previous bad response to treatment. However, the improvement observed in the GFR of the patients makes us think that the therapeutic potential of this intervention deserves to be discussed and further studied. Finally, we want to point out, we believe the way GF evolves to be a lead element to help us recognize the risk of running into chronification.

## 5. Conclusions

We may conclude that this parent-focused psychotherapy intervention is able to elicit changes in reflective functioning of parents of patients with AN. Changes in reflective functioning may be related to favorable modifications observed in family dynamics. Changes in family dynamics are probably related to the improvement of global functioning, symptomatology, and the insight of the studied patients where there has been previously poor response to standard treatment.

## 6. Limitations

We acknowledge the results presented here are preliminary. We know that presenting a retrospective case series study with a small population is a limitation in fully understand a disorder as complex as AN. Further studies comprising on the one hand, a quantitative methodology with a prospective and controlled design, and on the other, a qualitative methodology that explores in depth the individual processes of change, could strengthen the results presented here.

## Figures and Tables

**Table 1:** Clinical picture of patients at three points.

Patient	At Admission	At Reference	At Follow-Up End
1	Compulsive exercise, dietary restriction, purging behaviors. BMI 17.9. Poor academic performance, lack of social relationships. No insight, GAF 60.	12 months after ST: Impairment of compulsive and restrictive behaviors despite weight gain, began anxiety symptoms. BMI 19.1. School dropout, social isolation, cognitive impasse. No insight, GAF 60.	12 months after reference: Attached to dietary plan, healthy exercise pattern, stopped purging behaviors, voluntarily reinitiated pharmacologic treatment. BMI 20.4. Completed high school and got in to university, good academic performance. Improved socialization. Insight, GAF 90.
2	Severe dietary restriction (1 meal/day), purging behaviors, accumulation of rotten food, self-mutilation, suicide attempt. BMI 14.7. Poor academic performance, bad family relationship. No insight, GAF 50.	5 months after ST: Impairment of restrictive despite weight gain, remain self-mutilation behavior. BMI 18.2. School dropout, impaired family relationship, cognitive impasse. No insight, GAF 40.	18 months after reference: Attached to dietary plan, stopped purging behavior, self-mutilation disappeared, voluntarily reinitiated pharmacologic treatment. BMI 21. Back to school, improved family relationship. Insight, GAF 90.
3	Compulsive exercise, dietary restriction, insomnia, dyspnea, amenorrhea. BMI 17.4. Poor academic performance, bad relationship with her father. No insight, GAF 50.	4 months after ST: Reluctance to adhere to treatment, amenorrhea despite weight gain, symptoms of depression, suicidal ideation. BMI 19. Impaired relationship with her father and family, cognitive impasse. No insight, GAF 50.	12 months after reference: Stopped compulsive exercise, attached to treatment and dietary plan, recovered menstruation, voluntarily reinitiated pharmacologic treatment. BMI 21. Completed a university degree, got a job, living independently. Improved family relationship. Insight, GAF 80.
4	Dietary restriction, fasting. BMI 13.4. Poor academic performance, bad relationship with her mother. Bad social functioning. No insight, GAF 50.	7 months after ST: Without clinical improvement, school dropout, symptoms of depression, BMI 14.2. School dropout, impaired family and social functioning, cognitive impasse. No insight, GAF 40.	18 months after reference: Attached to dietary plan, stopped compensatory behavior, voluntarily reinitiated pharmacologic treatment. BMI 19.1. Improved relationship with her mother, got in to university with good academic performance. Insight, GAF 80.
5	Dietary restriction, obsessive caloric counting and weight quantification, severe insomnia, symptoms of anxiety. BMI 12. Bad social functioning, bad family relationship, no attending to school. No insight, GAF 40.	12 months after ST: Dietary restriction despite weight gain, impairment obsessive weight quantification and insomnia. BMI 18. Impaired social and family functioning, cognitive impasse. No insight, GAF 40.	12 months after reference: Attached to dietary plan, without compensatory behaviors, clinical improvement of anxiety. BMI 23. Completed a university degree, got a job, living independently, got married, had children. Insight, GAF 90.
6	Dietary restriction, compulsive exercise, symptoms of anxiety, obsessive ideas related to body image. BMI 15.6. Not attending school, quits every job he gets. Bad family relationship. No insight, GAF 60.	5 months after ST: Impairment of restrictive and compensatory behaviors despite weight gain, impairment in anxiety symptoms. BMI 21. Impairment of social and family functioning, cognitive impasse. No insight, GAF 50.	12 months after reference: Attached to dietary plan, stopped compensatory behaviors, clinical improvement of affective symptoms. BMI 25. Back to school, entered university, kept a stable job. Improvement in family relationship. Insight, GAF 90.
7	Dietary restriction, obsessive contamination ideas related to food, use of laxatives, caloric counting, fasting. BMI 14.5. Bad social, academic, and family functioning. No insight, GAF 40.	8 months after ST: Without clinical improvement in purging behaviors, amenorrhea despite weight gain, BMI 19.5. Impairment of social, academic, and family functioning, cognitive impasse. No insight, GAF 40.	12 months after reference: Attached to dietary plan, stopped compensatory behaviors, remission of obsessive ideas, recovered menstruation, voluntarily reinitiated pharmacologic treatment. BMI 25.7. Improved family and social functioning, she completed a university degree abroad. Insight, GAF 80.
8	Compulsive exercise, dietary restriction, obsessive ideas of food restriction, psychotic symptoms. BMI 15.8. Bad family and social functioning, neither attending school nor had a job. No insight, GAF 30.	5 months after ST: Despite weight gain dietary restriction remains. Without changes in obsessive ideas. BMI 18.4. Without changes in social, academic, and family functioning, cognitive impasse. No insight, GAF 30.	12 months after reference: Attached to dietary plan, remission of psychotic and obsessive symptoms. BMI 20.8. Entered university, got a stable job, improved family relationship, started a romantic relationship. Insight, GAF 80.
9	Dietary restriction, compulsive exercise, obsessive ideas about weight and body image, symptoms of depression. BMI 18.5. Bad social functioning, unable to keep a stable job, still living at her parents’ house. No insight, GAF 60.	16 months after ST: Despite weight gain, she was hospitalized for clinical impairment. Without improvement in depressive symptoms. BMI 20. Social isolation, remained without a job, cognitive impasse. No insight, GAF 40.	18 months after reference: Attached to dietary plan, stopped compensatory behavior, remission of obsessive and depressive symptoms, voluntarily reinitiated pharmacologic treatment. BMI 29. Kept a stable job for the first time (more than a year in the same place), living independently. Insight, GAF 80.
10	Dietary restriction, excessive water intake, counting calories, amenorrhea. BMI 16.8. School dropout, bad family relationship. No insight, GAF 40.	8 months after ST: Without clinical improvement, started abusing of alcohol. Despite weight gain, she was hospitalized. BMI 18. Neither attends school, nor has a job. Without change in family and social functioning, cognitive impasse. No insight, GAF 30.	18 months after reference: Stopped compensatory behaviors, with better attachment to dietary plan, still presenting episode of food restriction though. BMI 18.3. Back to school, finished high school and entered university, kept stable friendships, started a romantic relationship for 1st time, improvement in family relationship. Insight, GAF 90.
11	Dietary restriction, purge behaviors, compulsive exercise, amenorrhea, symptoms of depression, BMI 16.2. Social isolation, school dropout, without a job, bad family relationship. No insight, GAF 40.	5 months after ST: Without clinical improvement, amenorrhea despite weight gain, BMI 19.7. Impairment of family relationship, still not attending school, social isolation, cognitive impasse. No insight, GAF 40.	18 months after reference: Attached to dietary plan, stopped compensatory behaviors, recovered menstruation, remission of depressive symptoms. BMI 21.9. Finished school, got a stable job, started a stable romantic relationship, improvement of family relationship. Insight, GAF 90.
12	Dietary restriction, excessive exercise, use of laxatives, two suicidal attempts, amenorrhea, alcohol abuse, BMI 14.4. Bad family relationship, poor academic performance. No insight, GAF 30.	8 months after ST: Despite having stopped excessive exercise and gained weight, she was twice hospitalized for alcohol abuse. Still using laxatives, with amenorrhea. BMI 16.5. Without improvement in family and academic functioning, physical aggressiveness towards her parents, cognitive impasse. No insight, GAF 40.	6 months after reference: Attached to dietary plan, stopped purging behaviors. Stopped alcohol consumption. Without suicidal ideation, voluntarily reinitiated pharmacologic treatment. BMI 25. She entered university, got a scholarship abroad. Got a job, improvement in family relationship. Insight, GAF 90.
13	Dietary restriction, purge behaviors, accumulation of rotten food, compulsive exercise, suicidal ideation amenorrhea. BMI 11.5. Poor academic performance, bad family relationship. No insight, GAF 40.	6 months after ST: Despite weight gain she continued with purging and doing compulsive exercise. No changes in suicidal ideation and amenorrhea. BMI 18.5. Impairment in family relationship, still poor academic performance, cognitive impasse. No insight, GAF 50.	8 months after reference: Attached to dietary plan, stopped compensatory behaviors, without suicidal ideation, recovered menstruation, voluntarily reinitiated pharmacologic treatment. BMI 21.2. Finished school, entered university, got a job, improvement in family relationship. Insight, GAF 80.
14	Dietary restriction, obsessive ideas of control about food groups, excessive water intake, amenorrhea, suicidal ideation, self-mutilation, BMI 16.3. Bad family relationship, poor academic performance. No insight, GAF 30.	8 months after ST: Impairment of self-mutilation, without clinical improvement despite weight gain, BMI 18. Increased family conflicts, school dropout, social isolation, cognitive impasse. No insight, GAF 50.	6 months after reference: Attached to dietary plan, stopped food restriction and obsessive ideas about food, recovered menstruation, voluntarily reinitiated pharmacologic treatment. BMI 21. Improvement of family relationship and social functioning. She finished high school and entered university, started a romantic relationship. Insight, GAF 90.

ST = This refers to the moment when patients have undergone the treatment algorithm without a good response. The complete process takes at least 6 months, for those cases with a good response.

## Data Availability

The data presented in this study are available on request from the corresponding author. The data were obtained from the clinical records of the Clinic of Eating Disorders, of the National Institute of Psychiatry, Ramon de la Fuente Muñiz, so they are not publicly available due to privacy reasons.
